# Data-Driven Techniques for Low-Cost Sensor Selection and Calibration for the Use Case of Air Quality Monitoring

**DOI:** 10.3390/s22031093

**Published:** 2022-01-31

**Authors:** Rameez Raja Kureshi, Bhupesh Kumar Mishra, Dhavalkumar Thakker, Reena John, Adrian Walker, Sydney Simpson, Neel Thakkar, Agot Kirsten Wante

**Affiliations:** 1 Faculty of Engineering and Informatics, University of Bradford, Bradford BD7 1DP, UK; krameezr@bradford.ac.uk (R.R.K.); b.mishra@bradford.ac.uk (B.K.M.); rjohn1@bradford.ac.uk (R.J.); 2 City of Bradford Metropolitan District Council, Bradford BD1 1HX, UK; adrian.walker@bradford.gov.uk (A.W.); sydney.simpson@bradford.gov.uk (S.S.); 3 IBM India Pvt Ltd., Kolkata 700156, India; neelthakkar@acm.org; 4 IVL Swedish Environmental Research Institute, 114 28 Stockholm, Sweden; agot.watne@ivl.se

**Keywords:** Low-Cost Sensor (LCS), calibration, data-driven techniques, drift analysis, air quality

## Abstract

With the emergence of Low-Cost Sensor (LCS) devices, measuring real-time data on a large scale has become a feasible alternative approach to more costly devices. Over the years, sensor technologies have evolved which has provided the opportunity to have diversity in LCS selection for the same task. However, this diversity in sensor types adds complexity to appropriate sensor selection for monitoring tasks. In addition, LCS devices are often associated with low confidence in terms of sensing accuracy because of the complexities in sensing principles and the interpretation of monitored data. From the data analytics point of view, data quality is a major concern as low-quality data more often leads to low confidence in the monitoring systems. Therefore, any applications on building monitoring systems using LCS devices need to focus on two main techniques: sensor selection and calibration to improve data quality. In this paper, data-driven techniques were presented for sensor calibration techniques. To validate our methodology and techniques, an air quality monitoring case study from the Bradford district, UK, as part of two European Union (EU) funded projects was used. For this case study, the candidate sensors were selected based on the literature and market availability. The candidate sensors were narrowed down into the selected sensors after analysing their consistency. To address data quality issues, four different calibration methods were compared to derive the best-suited calibration method for the LCS devices in our use case system. In the calibration, meteorological parameters temperature and humidity were used in addition to the observed readings. Moreover, we uniquely considered Absolute Humidity (*AH*) and Relative Humidity (*RH*) as part of the calibration process. To validate the result of experimentation, the Coefficient of Determination (*R*^2^), Root Mean Square Error (*RMSE*), and Mean Absolute Error (*MAE*) were compared for both *AH* and *RH*. The experimental results showed that calibration with *AH* has better performance as compared with *RH*. The experimental results showed the selection and calibration techniques that can be used in designing similar LCS based monitoring systems.

## 1. Introduction

Low-Cost Sensors (LCSs) are changing the conventional way to monitor and measure instances in real-time with the help of micro-scale sensing techniques [1]. The use of LCSs has benefits in terms of cost-effectiveness, compactness, and portability which make these devices an efficient alternative against the high-cost monitoring systems [2]. For example, building LCS-based air quality monitoring devices (use case used in this study) to deploy against high-cost air quality monitoring stations in a city is more feasible in terms of high spatio-temporal and instantaneous data monitoring to the user at any specific location. Additionally, LCSs have appeared as an economical substitute for high-cost sensors devices in many applications including air quality monitoring (Indoor–Outdoor), flood monitoring, and observing health status. In recent years, with the enhancement in sensor technology, there have been many alternative sensors to perform the same tasks while developing any LCS-based applications. However, there has not been a universal single type of LCS implementation as the LCSs have different working principles such as electrochemical, optical particle counters (OPC), non-dispersive infra-red (NDIR), metal-oxide-semiconductor, or solid-state microsensors designed to monitor air pollutants [3,4,5]. This diversity in working principles of sensors adds complexity to the process of LCS selection while building the monitoring systems using LCS-based devices. Apart from the working principle of LCS devices, meteorological parameters such as temperature and humidity make LCS data unreliable and less accurate when they have been used in an open environment [6,7]. Furthermore, LCSs are monitoring various components from the environment according to their function which can cause sensitivity issues [8,9]. For example, when the LCS is used to monitor air pollutants, the sensor also responds more to other gas compounds in addition to the actual gas detection [10]. Moreover, LCSs have characteristics that cannot give a stable performance over a certain period due to drift of sensitivity and ageing which can cause increased data inaccuracy [9,11]. Apart from the issues discussed earlier, LCSs also often face challenges to detect measured levels below a point where it would not be able to differentiate between sensor noise and actual sensed values in the environment [9,12,13]. This is because the sensor is designed in a range called the dynamic boundary. When the sensor faces the actual data level near or below the dynamic boundary, it often fails to monitor the data accurately. To overcome this, it is a prerequisite to calibrate LCSs before the on-field application is required [14]. In addition to these challenges, LCS data quality and their consistency under the same environment are other factors that make the LCS-based applications even more complex.

Considering the lower feasibility of high-cost air quality monitoring sensors to deploy at many strategic locations within a small area, LCS-based devices become a feasible solution for many applications. However, the use of LCSs brings forth its challenges in terms of sensor selection, quality of data and accuracy of measurement [9,15]. There have been different applications that have used LCSs for real-time monitoring, but these applications do not talk about the sensor selection and their calibration process [3,5,8,9,10,16]. Furthermore, the market availability of sensors in different forms to measure the same component adds a challenge to LCS selection. To our best knowledge, there have been challenges in terms of how to select, calibrate, and build LCS-based devices considering twin factors of sensors’ availability and data quality. To improve the data quality issue, calibration methods have been used to improve LCS-based devices’ performances. Over the years, there have been different calibration methods such as Multiple Linear Regression (MLR) [5,17], Random Forest (RF) [18,19], Support Vector Regression (SVR) [3], and Artificial Neural Networks (ANN) [20] have been used to improve data quality. Finding an accurate calibration method for the LCS selection based on the LCS deployment environment has made the calibration task further complex as various LCSs have different working principles and configurations [9]. Although different calibration methodologies have been applied to improve data quality to the LCS-based monitoring applications, there is a need for a more precise and comparable approach on how to calibrate LCSs to ascertain data quality assurance with respect to industrial scale, high-cost, high-quality reference data. Since the calibration is dependent on the types of LCS-based devices, applications, deployment environment, and meteorological parameters, there is a need for a well-defined methodology for sensor calibration.

In this paper, a data-driven LCS calibration methodology was presented to address the challenges of LCS-based device building for an application. In this proposed methodology, at first, candidate LCS selection was applied based on literature reviews and sensors’ market availability. Data-driven statistical analyses [21] give the confidence to select the most consistent LCSs for building LCS-based devices to improve data quality. In this paper, considering the air quality monitoring application, a comparative analysis was explored among the widely used calibration methods by applying Absolute Humidity (*AH*) and Relative humidity (*RH*) along with temperature and measured pollutants. Four different calibration methods, (i) Multivariate Linear Regression, (ii) Multi-Layer Perceptron (MLP), (iii) Convolution Neural Network (CNN), and (iv) Random Forest (RF) were compared with both *AH* and *RH* as calibration parameters to find the best-suited calibration method for the selected LCS in a real-world use case application in two EU projects. The calibration of selected LCSs was accomplished with the reference of a high-cost air quality monitoring station from Urban Observatory, Sheffield (https://urbanflows.ac.uk/, accessed on 1 March 2021). Among different calibration models, the RF model has better performance in terms of the coefficient of determination, root mean square error, and mean absolute error. In addition, it was observed that calibration accuracy has better performance when *AH* was used over *RH* as one of the calibration parameters.

The rest of the paper is organised as follows. In Section 2, a comprehensive review of LCSs and LCS-based applications is presented. In Section 3, the data-driven calibration techniques are presented. In Section 4, the experimental result analysis and discussion are presented. In Section 5, the paper is completed with a conclusion along with future work.

## 2. Literature Review

With the increase in the availability of micro-sensor technology in recent years, the use of LCS-based devices has been boosted in monitoring and measurements applications with wider spatial coverage in different domains [22]. Comparing LCSs with high-cost monitoring stations, LCSs are less expensive and easy to access and deploy. Though, the data from LCSs are generally less reliable with low quality [9,15,23]. Predominantly, the data collected from LCS-based devices are easy to handle, process, and can be analysed by experts allowing sharing the monitoring outcomes with the public or stakeholders to spread awareness and other purposes [1]. There has been a wide range of application domains such as air quality [16], road traffic [24], water quality [25] and human health [26] which makes the LCS-based devices more demanding. Castell et al. [10] evaluated the performance of the LCS (AQMesh) and analysed data quality. They also listed several funded applications based on LCS-based applications such as OpenSense (https://gitlab.ethz.ch/tec/public/opensense, accessed on 15 February 2021), Everyaware (http://www.everyaware.eu/, accessed on 20 February 2021), Citi-Sense-MOB and Citi-Sense [27] where the devices mount on vehicles or any stationary location and start sending real-time data to their web platform from eight European cities for further processing. These devices have individual gas sensors which cost around EUR 20–100 each and the whole monitoring device cost range is EUR 500-5000 approximately. Chojer et al. [16] reviewed various works from 2012 to 2019 based on LCS-based monitoring systems and finalised the most relevant 35 research applications with meteorological parameters such as temperature and *RH*. Additionally, this study has shown that out of 35 applications, only 10 applications were focused on sensor performance including validation, calibration, and testing. The main benefit of LCS-based applications is their affordability and availability in the market [28]. Kumar et al. [29] showed the benefits of LCS-based applications in terms of accessing real-time data, increased spatial resolution, reduced uncertainty, identification of emitting sources from indoor activities, and health benefits compared with the traditional monitoring systems. This work also highlighted some challenges such as data quality and performance evaluation for LCS-based device development.

Some of the studies [30,31,32] showed that neither temperature nor *RH* has any influence on LCS performance. Zou et al. [33] experimented with eight different low-cost *PM* sensors to find the relation between temperature and humidity in a lab environment and found that the temperature does not have any significant effect on LCS functioning. Additionally, their study highlighted that the *RH* in the range of 10–90% may affect the magnitude of the sensor’s output and it can be improved through calibration. Zamora et al. [34] show that the meteorological parameter *RH* has a significant impact on LCS performance. Similarly, Jayaratne et al. [35] have experimented with different Particulate Matter (*PM*) sensors and found that if *RH* exceeds 75% then it affects the sensor’s performance adversely. Laurent et al. [36,37] used *AH* instead of *RH* and temperature for the LCS calibration process. This study raised the significance of environmental parameters and working principles of LCSs, which required more sophisticated calibration efforts for data reliability.

Sensor calibration is the method of adjustment performed on the sensor to make the functionality of the sensor as accurate as possible and error-free. Maag et al. [9] surveyed various calibration methods and categorised calibration types into two parts: pre-deployment calibration (calibration model) and post-deployment calibration (network recalibration strategies). Additionally, this literature review listed an overview of sensor calibration models classified as follows: i. Offset and Gain Calibration; ii. Temperature and Humidity Correction; and iii. Sensor Array Calibration. Offset and gain calibration gather calibration errors due to uncertain boundaries and discard any possible non-linear responses. Temperature and humidity correction extends the recorded values with existing values to calibrate the LCS. Sensor array calibration is an extension of temperature and humidity correction where it applies interfering gases and environmental factors. Sensor calibration can be done either in a controlled environment or an uncontrolled environment. A controlled environment implies calibration of the sensor either with high-end instruments or with already calibrated sensors. Whereas, an uncontrolled environment implies that sensor parameters are regulated according to other sensors because it cannot measure the data in a controlled environment which can lead to erroneous data monitoring [36,38].

In general, the monitoring stations with high-cost equipment are deployed at static locations. The high-cost devices are reliable but infeasible to deploy in different locations [10]. However, there is always a trade-off between high cost and generally high-fidelity devices with low-cost and often low-fidelity devices. This raises an issue when selecting appropriate LCSs that can minimise this trade-off and provide more reliable data. All applications based on LCSs have common problems that are data reliability and data quality [5,18]. Maag et al. [9] surveyed LCS problems and found that LCS data quality can be influenced and relied upon by several factors such as sensor types and working principles, meteorological parameters, low sensitivity, and sensor consistency. Kotsev et al. [39] explain the approaches for reliable data quality from LCSs and mention some of the known parameters which can affect electrochemical sensor responses such as temperature, humidity and cross-sensitivity.

As a solution to address all of these challenges of LCSs, sensor calibration is required [40,41]. Some of the calibrations have been conducted in a lab environment such as the research of Wang et al. [42] who calibrated LCS *PM* in lab conditions whereas Spinelle et al. [37] applied on-field calibration of LCSs against a reference station. Choosing a calibration model depends upon certain parameters such as the type of sensor, type of phenomena of the device, resources required for that device, storage, computation, and communication capabilities of those sensors [38]. Multivariate Linear Regression (MLR) has been widely used for calibration [5,17]. For the calibration using MLR, two or more covariates are mainly used to get the targeted variables outcome. The MLR model is easy to implement but has limitations as well since the MLR used a linear equation with coefficients based on some assumptions such as linearity, residual error, and co-linearity. Another model that has been also widely used is Random Forest (RF) [18,19]. The RF-based model improved the stability by randomly selecting several observations from the dataset during training leaving some of the datasets for testing the model. The predictions are based on the mean of the results coming from a number of trees. However, the tree size grows with the increase in the dataset size, which increases time complexity while training the model. Support Vector Regression (SVR) appeared as another method used for the calibration [3]. An SVR-based calibration model uses kernel functions to train the model with the given datasets. SVM generates an optimal hyperplane to distinguish different classes to predict the outcomes. However, the SVM-based model required users to define the number of support vectors. Artificial neural networks (ANN) [20] are also among the commonly used calibration models. ANN-based models are mostly used when datasets have noise. However, the ANN training requires a number of iterations with certain user-defined parameters such as the number of nodes, hidden layers, activation function, and weights. The performance of the ANN model depends on the user-defined parameters.

In these calibration processes, experts have used statistical calibration models as mentioned above. The selection of the right sensors based on the literature and market accessibility is also challenging due to the wide-ranging availability of LCSs. Williams et al. [43] provide a guidebook that can help with LCS selection, however, their studies suggested that the sensor selection fully relies on the user preferences based on the sensor manual. In a similar study [44], it has been explained that the sensor selection process fully depends upon the end-user and it is an application that can help to define the scope. Similarly, Sousan et al. [45] checked the consistency of *PM* sensors (Sharp GP sensors) using the average slope method where it has been found that calculating the average of multiple measurements over the large time-frequency can decrease the random noise and hence increase the data quality.

Going through the literature, it has been observed that many applications have been developed using LCSs. The literature also highlights the increase in LCS-based applications over the years. The applications areas of LCSs are not limited to only a few domains, rather LCS-based applications have been covered in wider domains suggesting that LCSs have been extensively used in recent years. The enhancement in the use of LCSs in different applications is due to the emergence of a wide range of LCSs and alternatives for similar tasks. From the literature, it has been also noted that there has been the use of different sensors in different applications which raises the challenge of sensor selection while developing LCS-based devices. With the appearance of more alternative LCSs for the same tasks, the sensor selection challenge will increase further in the coming years. Therefore, there is a need for the sensor selection strategy to build effective LCS-based applications. Data quality has been another concern that is argued in many LCS-based applications. Different calibration methods have been applied in different applications. However, how different meteorological parameters affect the calibration method and which calibration method is more efficient remains a challenging aspect as the calibration process efficiency depends on the parameters being used for calibration, the sensor working principles, and the application domain. In general, to build an effective LCS-based device for an application, sensor selection and calibration are crucial for making the LCS-based system more effective with higher data quality.

## 3. Data-Driven Sensor Calibration: Our Methodology

**Figure 1:**
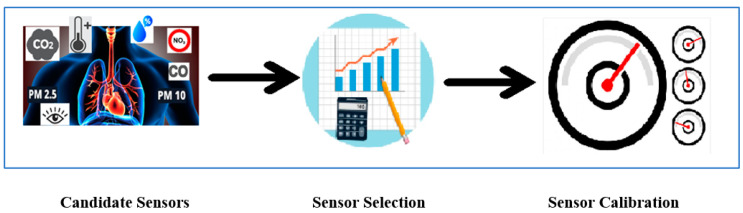
Process diagram for data-driven sensor calibration methodology to build air quality monitoring devices.

For the development of LCS devices, a data-driven sensor calibration methodology, as shown in Figure 1, was presented. At first, several sensors were selected as candidate sensors based on literature and market availability. From the literature, we enlisted widely used candidate sensors such as OPC-R1, PMS5003, *PM* Nova SDS011, and Particulate Matter Sensor SPS30 for *PM* monitoring, SGP30, CJMCU-611, and CU-1106 for CO_2_ monitoring and similarly for other air pollutants monitoring sensors. While deciding on the candidate sensors, we considered the sensor’s availability in the UK market and their suppliers. The suppliers were chosen based on the procurement criteria set by our organisation. Following this, we also explored the datasheets provided by the manufacturer and available libraries to support sensor implementation with programming. By applying these three factors, we shortlisted candidate sensors to examine for our use case. After that, sensors were selected based on the statistical analysis presented in our previous work [21]. In the next step, the calibration process for the selected sensors was applied to improve data quality in the selected use case.

**Use case:** Bradford district, UK, air quality (indoor and outdoor) monitoring is presented as the use case in this study. There have been more than 1500 air quality monitoring stations, such as Automatic Urban and Rural Network (AURN) (https://uk-air.defra.gov.uk/networks/network-info?view=aurn, accessed on 17 January 2021), deployed across the UK. These stations have installed large, expensive, and calibrated sensors devices that can monitor several air pollutants such as oxides of nitrogen (NOx), sulphur dioxide (SO_2_), particles (*PM*_10_ and *PM*_2.5_), carbon monoxide (CO), and ozone (O_3_). Additionally, in many cases, these stations are located away from traffic areas or a small distance from city centres which can put a limitation on this station’s coverage to monitor air quality. The limitation on coverage area from the high-cost monitoring stations in Bradford city allows deploying LCS-based devices as an effective alternative to monitor air quality (indoor-outdoor) across the city and also allows real-time exposure assessment from many locations. This work contributes to part of two EU projects, Smart Cities and Open data Reuse (SCORE) and LifeCritical. For both projects, an area of Bradford needs to be covered with sensors indoors and outdoors to support constant monitoring of air quality over a year. The focus is to have sufficient geographical coverage of this area to support a granular level of monitoring. The high-cost and immobile devices, used, for example, as part of AURN, is completely impractical. Hence, we needed to rely on the use of LCS-based devices for this purpose where the accuracy of monitoring is paramount in order to support analytics and policymaking.

### 3.1. Selection of Candidate Sensors

In the LCS-based use case, the aim is to build a reliable air quality monitoring system that can measure several pollutants such as PM, CO_2_, NO_2_, NH_3_, CO, and Volatile Organic Compounds (VOCs) as these pollutants are heavily dependent on various activities carried out by humans [46]. The pollutant concentration depends not only on the emissions of a pollutant but also on meteorological conditions like wind speed (WS), relative humidity (*RH*), and turbulence. The meteorological parameters and other atmospheric compounds can influence the sensor measurements. For the gas sensors, there can be a cross-sensitivity. In other words, the concentration of a particular pollutant measured by a sensor can be affected by the concentration of a different pollutant due to the measurement techniques. However, the cross-sensitivity of sensors was not considered when selecting the gas sensors for this study. We relied on the information provided by the manufacturer to measure particular gases. For the use case, the following candidate sensors, as listed in Table 1, were considered to measure air pollutants either outdoors or indoors. These candidate sensors were selected based on the existing studies and market availability [47,48,49].

### 3.2. Narrowing down the Selection of Low-Cost Air Quality Monitoring Sensors

The next challenge was to find the most feasible sensors from the candidate sensors. To achieve this, statistical analysis presented in our earlier work [21] was applied that gives the most feasible sensors among the candidate sensors. For example, to measure *PM*_2.5_ and *PM*_10_, three different sensors SDS011, PMS5003, and OPC-R1 were compared in the lab environment for 48 h to find the best feasible sensor among these three for *PM* measurements. If the sensors from the same type and same manufacturer are not consistent among themselves when they are exposed to the same environment, then they need to be discarded from consideration in the next step of calibration.

All selected LCSs have working limitations, mentioned in the manufacturer’s datasheet, e.g., SDS011 has a particle measurement range of 0.00–999.99 μg/m^3^. In the lab experimentation, we considered the general case scenarios that reflect that all sensors’ ranges’ fall under the general working environment.

### 3.3. Sensor Calibration

**Figure 2:**
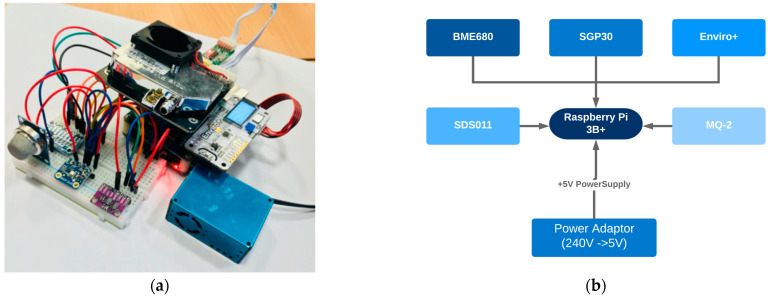
(**a**) Final LCS-based AQ monitoring device using RPi 3B+. (**b**) Block diagram of the final device with Raspberry Pi 3B+ and other LCS components.

After implementation of previous steps, BME680, SGP30, Enviro+, MQ-2, and SDS011 sensors were selected. These sensors were used to build an Air Quality (AQ) monitoring device for measuring air pollutants, as shown in Figure 2. For computational and connectivity purposes, selected sensors were assembled with Raspberry Pi 3B+ (RPi) which gives remote access control of the whole device and sends data to the cloud-based web server for data storage, analysis, and visualisation.

**Figure 3:**
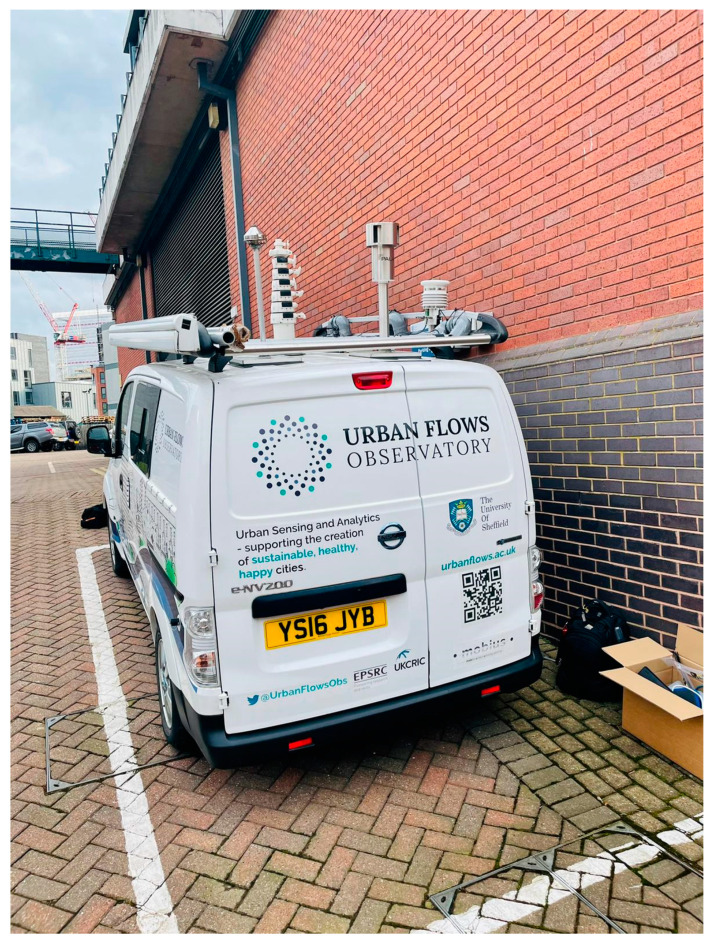
LCS-based AQ monitoring devices using RPi 3B+ at Urban Observatory, Sheffield Site.

After building the air quality monitoring LCS-based device, data quality aspects were applied. Studies have argued that [5,9,18] sensor calibration is required to increase the data quality of LCS-based systems. Considering this, from the calibration point of view, the two AQ devices were deployed at “The Urban Flows Observatory (https://urbanflows.ac.uk/, accessed on 1 March 2021), Sheffield” (Figure 3) for one month. Both the devices had all selected sensors that monitor air pollutants such as CO_2_, NO_2_, CO, NH_3_, TVOC, and *PM* (*PM*_2.5_ and *PM*_10_). Additionally, these devices had sensors that can measure meteorological parameters (temperature and humidity). In this paper, the calibration methods for *PM*_2.5_ and *PM*_10_ are presented. For *PM*_2.5_ and *PM*_10_ data calibration, SDS011 *PM* sensors were calibrated against the “high-end Palas Fidas 200” instrument which was installed at the remote van, as shown in Figure 3, by Sheffield City Council. This station monitors data at the 30 min interval whereas our AQ devices monitor data at every 10 min interval. Data pre-processing was applied to the AQ data to convert into 30 min data using the mean value of three 10 min readings. All the measured data were received from the AQ devices in every 10 min time interval that was sent to the cloud-based web server (http://smartbradford.co.uk:7201/, accessed on 12 January 2021) and also stored in the AQ devices in CSV (Comma-Separated Values) format.

Different calibration models were experimented with to compare and select the most accurate model. For example, we took *PM*_2.5_ and *PM*_10_ data for experimental purposes and undertook experiments on four calibration models: Multivariate Linear Regression (MLR), Multi-Layer Perceptron (MLP), Convolution Neural Network (CNN), and Random Forest (RF). The literature has argued that *AH* and *RH* act differently with different LCSs in the calibration process. Mead et al. [50] show that the *RH* greatly depends on temperature, therefore, fluctuations can be observed in *RH* throughout the day. In contrast, *AH* is observed to be constant as it is independent of temperature. Due to this factor, the calibration process was adapted based on *AH* as the corrections were constant and linear based on per unit change in *AH*. Piedrahita et al. [51] observed that temperature has a significant impact on this sensor signal response, but the impact of *AH* is lower on the signal response as it has been observed to be almost constant, contradictory to the *RH* impact on sensor signal response. However, they still consider *AH* in calibration modelling to improve the model performance. Additionally, some of the studies [52,53] show that temperature and humidity have a non-linear relationship with particle concentrations. Research also shows that *PM*_2.5_ and *PM*_10_ values have a positive correlation with *RH* but a negative correlation with temperature and *AH* [37]. Considering these previous studies, in this work, temperature and humidity along with the pollutant were applied for the calibration. For humidity as a factor in calibration, both *AH* and *RH* were examined to determine which among these two humidity measures gives better results for the data quality.

To find *AH*, we used the Clausius Clapeyron equation [54] as shown in Equation (1),

(1) $$AH\;=\;\frac{6.112*e^{\left[\frac{17.67*T}{T+243.5}\right]}*RH*2.1674}{273.15+T}$$

where,

*T* = Temperature (*C)
*RH* = Relative Humidity (%)
*e* = Exponential function

Using Equation (6), *AH* was calculated based on the two observations *T* and *RH* coming from the BME680 sensor and the exponential function. This *AH* was used as one of the parameters for the calibration process.

(2) $$R^{2}=\frac{SS_{RES}}{SS_{TOT}}=1-\frac{\sum_{i}^{}{\left(y_{i}-{\hat{y}}_{i}\right)}^{2}}{\sum_{i}^{}{\left(y_{i}-\bar{y}\right)}^{2}}$$

(3) $$RMSE=\sqrt{\sum_{i=1}^{n}\frac{{\left({\hat{y}}_{i}-y_{i}\right)}^{2}}{n}}\;$$

(4) $$MAE=\sum_{i=1}^{n}\frac{{\left|\left({\hat{y}}_{i}-y_{i}\right)\right|}^{}}{n}$$

where

*R*^2^ = R-squared.
*RMSE* = Root Mean Square Error.
*MAE* = Mean Absolute Error.
*SS_RES_* = Residual sum of squared errors of our regression model.
*SS_TOT_* = Total sum of squared errors.
$y_{i}$ = Observed value from our kit.
${\bar{y}}_{i}$ = Mean value of pollutants value from our kit.
${\hat{y}}_{i}$ = Values predicted by the model.
*n* = Number of observations.

For comparative analysis between *RH* and *AH*, we experimented with selected models and analysed their impacts on the results. Additionally, for model evaluation, the following statistical measures were used as shown in Equations (2)–(4). Using these equations, performance measures such as *R*^2^, *RMSE*, and *MAE* were calculated using observed values ($y_{i}$) recorded using our LCS-based device and (${\bar{y}}_{i}$) recorded as a mean value of pollutant values from our devices. *R*^2^ was calculated as a ratio of the residual sum of squared errors (*SS_RES_*) of our regression model and the total sum of squared errors (*SS_TOT_*).

#### 3.3.1. Multivariate Linear Regression

Multivariate Linear Regression (MLR) is one of the widely used calibration methods applied for two or more independent variable dependencies with one single targeted variable by adjusting coefficients in linear equations, as represented in Equation (5) [55].

*y_i_* = *a_p_* * *x_ip_* + … + *a*_1_ * *x*_*i*1_ + *a*_0_ + *z_i_* (5)

Equation (5) is the generalised representation of MLR where *a_p_*,* a*_1_, and *a*_0_ are coefficients, *x_ip_*, *x*_*i*1_ are dependent variables, *z_i_* is constant, and *y_i_* is the calibrated targeted variable. In this study, the MLR model was applied for the selected sensors using Equations (6) and (7).

*ŷ_ref_* = *b*_0_ + *b*_1_ * *T* + *b*_2_ * *PM_raw_* + *b*_3_ * *AH* (6)

*ŷ_ref_* = *b*_0_ + *b*_1_ * *T* + *b*_2_ * *PM_raw_* + b_3_ * *RH* (7)

where

*ŷ_ref_* = reference data from Palas Fidas 200, Sheffield.
*b*_0_, *b*_1_, *b*_2_, and *b*_3_ = Regression coefficients.
*T* = Temperature (*C), *RH* = Humidity (%) from the BME680 sensor.
*AH* = Absolute Humidity (g/m^3^).
*PM*_2.5*raw*_ = Mean *PM* data (from SDS011).

**Figure 4:**
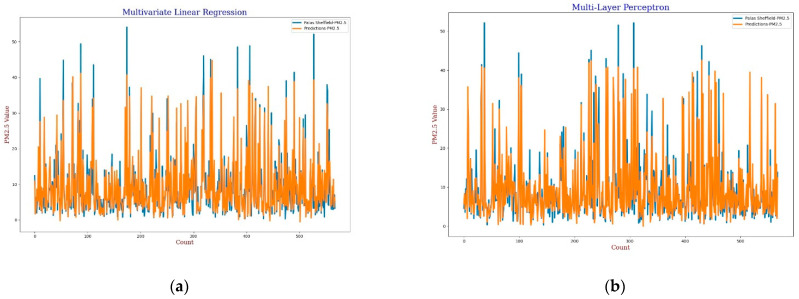
(**a**) MLR calibration plot for *PM*_2.5_ using *AH*. Blue colour represents the reference data and orange colour represents the calibrated data; (**b**) MLR calibration plot for *PM*_2.5_ using *RH*. Blue colour represents the reference data and orange colour represents the calibrated data.

**Figure 5:**
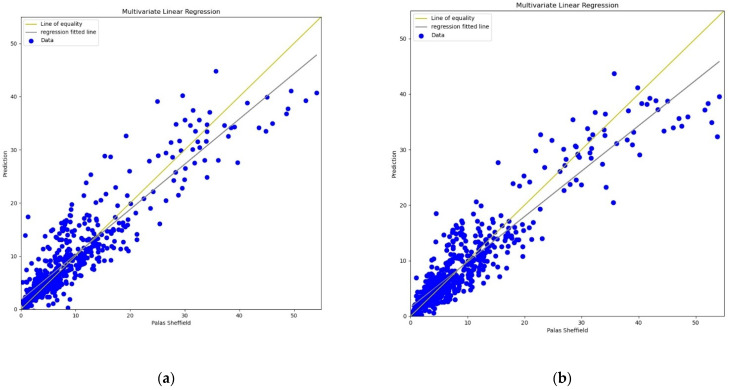
(**a**) MLR calibration scatter plot for *PM*_2.5_ with the line of equality, regression fitted line, and the calibrated data using *AH*; (**b**) MLR calibration scatter plot for *PM*_2.5_ with the line of equality, regression fitted line, and the calibrated data using *RH*.

**Figure 6:**
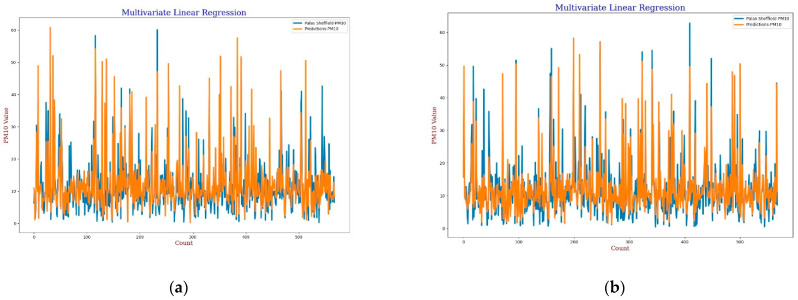
(**a**) MLR calibration plot for *PM*_10_ using *AH*. Blue colour represents the reference data and orange colour represents the calibrated data; (**b**) MLR calibration plot for *PM*_10_ using *RH*. Blue colour represents the reference data and orange colour represents the calibrated data.

**Figure 7:**
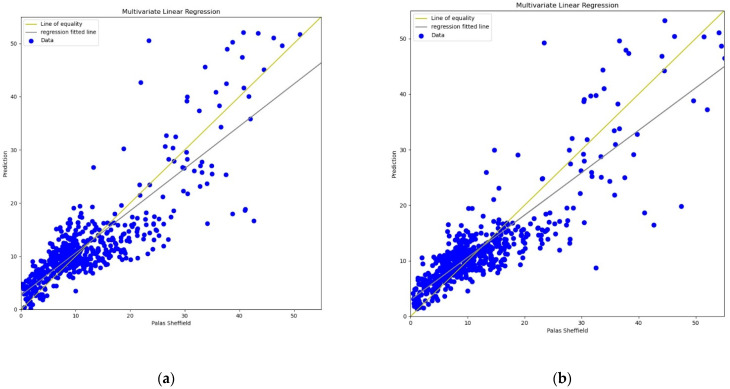
(**a**) MLR calibration scatter plot for *PM*_10_ with the line of equality, regression fitted line, and the calibrated data using *AH*; (**b**) MLR calibration scatter plot for *PM*_10_ with the line of equality, regression fitted line, and the calibrated data using *RH*.

For the calibration analysis, line and scatter plots were presented for both *PM*_2.5_ and *PM*_10_ as shown in Figure 4, Figure 5, Figure 6 and Figure 7. Figure 4 and Figure 6 present the line plots for *PM*_2.5_ and *PM*_10_, respectively, whereas Figure 5 and Figure 7 shows scatter plots for *PM*_2.5_ and *PM*_10_, respectively. Further detailed statistical analysis was also undertaken (presented in Section 4) to validate the graphical analysis. From the line plots, for *PM*_2.5_ and *PM*_10_, it can be observed that calibrated values are closer to the reference data when *AH* was used. From the scatter plots, it can be also observed that the regression fit line is closer to the line of equality when *AH* was used for the calibration in comparison to the *RH* for both *PM*_2.5_ and *PM*_10_. Analysis of these plots infers that *AH* has better performance than *RH* in the calibration process.

#### 3.3.2. Statistical Approaches

**Figure 8:**
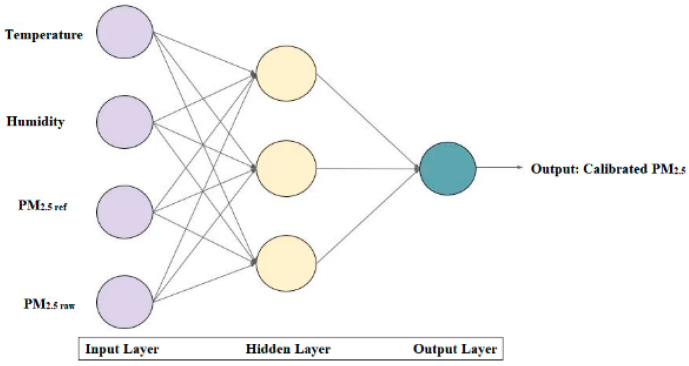
Block diagram of the Multi-Layer Perceptron model.

Multi-layer perceptron (MLP) is a forward-structured Artificial Neural Network that operates on sets of input vectors to give output with a set of output vectors. MLP is one of the efficient calibration methods that has been applied in many problems [56]. The Multi-Layer Perceptron model, as shown in Figure 8, was designed as the second calibration model. In the input layer, four parameters, temperature, humidity (*AH* and *RH*), *PM_ref_*, and *PM_raw_* were applied and the calibrated PM value was obtained at the output layer. The same dataset used for MLR was used for MLP as well. The model was designed as a sequential model with relu activation function, Adam optimiser, and mean square error as a loss function with 2000 epochs for training.

**Figure 9:**
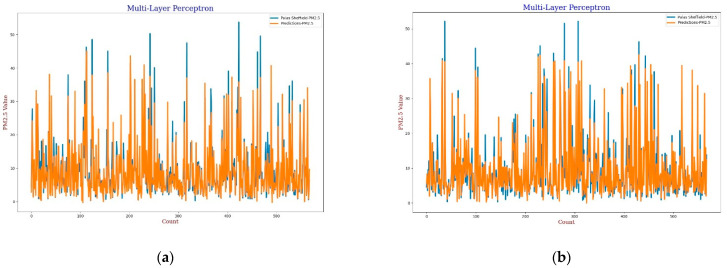
(**a**) MLP calibration plot for *PM*_2.5_ using *AH*. Blue colour represents the reference data and orange colour represents the calibrated data; (**b**) MLP calibration plot for *PM*_2.5_ using *RH*. Blue colour represents the reference data and orange colour represents the calibrated data.

**Figure 10:**
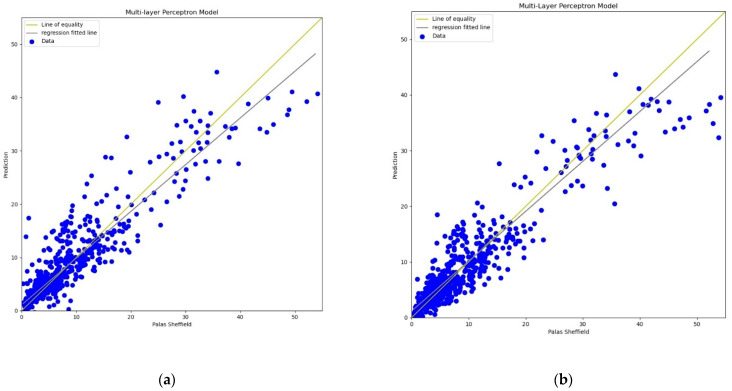
(**a**) MLP calibration scatter plot for *PM*_2.5_ with the line of equality, regression fitted line, and the calibrated data using *AH*; (**b**) MLP calibration scatter plot for *PM*_2.5_ with the line of equality, regression fitted line, and the calibrated data using *RH*.

**Figure 11:**
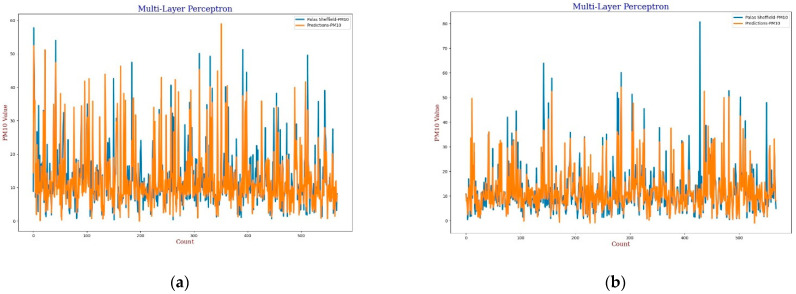
(**a**) MLP calibration plot for *PM*_10_ using *AH*. Blue colour represents the reference data and orange colour represents the calibrated data; (**b**) MLP calibration plot for *PM*_10_ using *RH*. Blue colour represents the reference data and orange colour represents the calibrated data.

**Figure 12:**
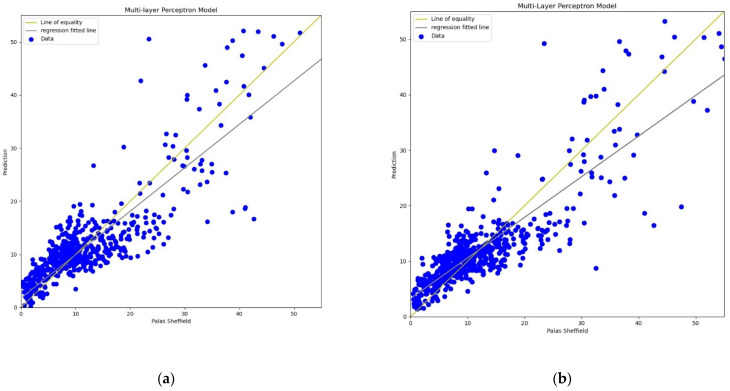
(**a**) MLP calibration scatter plot for *PM*_10_ with the line of equality, regression fitted line, and the calibrated data using *AH*; (**b**) MLP calibration scatter plot for *PM*_10_ with the line of equality, regression fitted line, and the calibrated data using *RH*.

For the MLP calibration analysis, line and scatter plots were observed for both *PM*_2.5_ and *PM*_10_ as shown in Figure 9, Figure 10, Figure 11 and Figure 12, where Figure 9 and Figure 11 present the line plots for *PM*_2.5_ and *PM*_10_, respectively, and Figure 10 and Figure 12 show scatter plots for *PM*_2.5_ and *PM*_10_, correspondingly. The same as MLR, the calibrated line plots for *PM*_2.5_ and *PM*_10_ are closer to the reference value when *AH* has been used. Similarly, in the scatter plots, the regression fit line is closer to the line of equality when *AH* has been used for the calibration in comparison to the *RH* for both *PM*_2.5_ and *PM*_10_. Analysis of these plots concludes that *AH* has better performance than *RH* in the calibration process in the case of MLP as well.

#### 3.3.3. Convolution Neural Network

**Figure 13:**
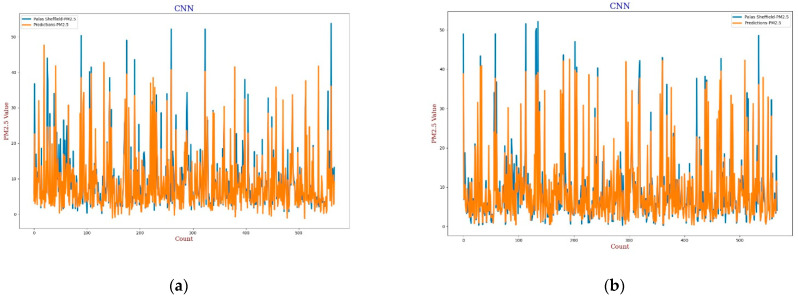
(**a**) CNN calibration plot for *PM*_2.5_ using *AH*. Blue colour represents the reference data and orange colour represents the calibrated data; (**b**) CNN calibration plot for *PM*_2.5_ using *RH*. Blue colour represents the reference data and orange colour represents the calibrated data.

**Figure 14:**
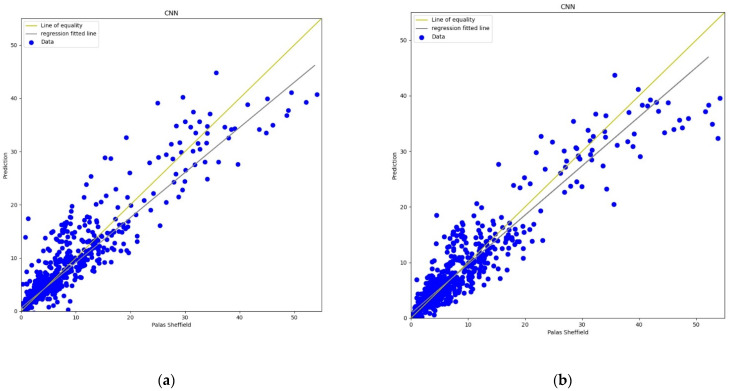
(**a**) CNN calibration scatter plot for *PM*_2.5_ with the line of equality, regression fitted line, and the calibrated data using *AH*; (**b**) CNN calibration scatter plot for *PM*_2.5_ with the line of equality, regression fitted line, and the calibrated data using *RH*.

**Figure 15:**
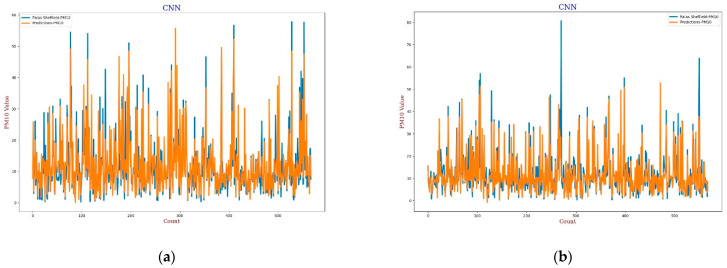
(**a**) CNN calibration plot for *PM*_10_ using *AH*. Blue colour represents the reference data and orange colour represents the calibrated data; (**b**) CNN calibration plot for *PM*_10_ using *RH*. Blue colour represents the reference data and orange colour represents the calibrated data.

**Figure 16:**
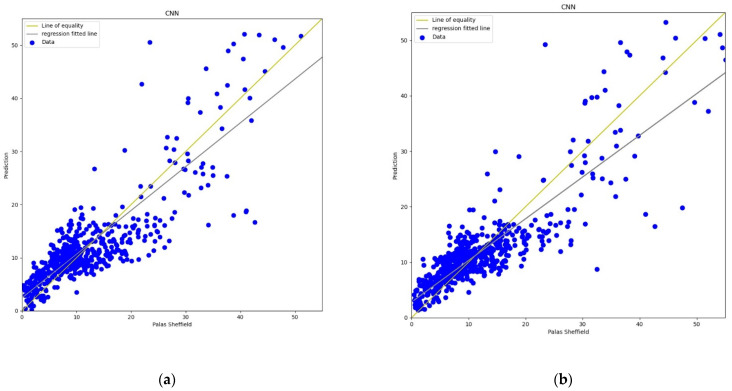
(**a**) CNN calibration scatter plot for *PM*_10_ with the line of equality, regression fitted line, and the calibrated data using *AH*; (**b**) CNN calibration scatter plot for *PM*_10_ with the line of equality, regression fitted line, and the calibrated data using *RH*.

Recently, CNN architectures have been used in various modelling scenarios of sequential data such as time series [57,58]. CNN has appeared as one of the most widely used calibration models as CNN can extract inherent information from the data set [59]. In the calibration, the same as the MLP model, the CNN model has four 3D inputs and reshape was applied that gives one output, two hidden convolutional layers with 64 filters each, and a window size of 2 was also defined for the CNN model. All layers were activated through the “relu” function with 2000 epochs support with the “adam” optimiser. The output in terms of line plots and scatter plots were analysed for both *AH* and *RH* as shown in Figure 13, Figure 14, Figure 15 and Figure 16. This model also has similar results in both plots that support better calibration performance for *AH* in comparison to *RH*.

#### 3.3.4. Random Forest

**Figure 17:**
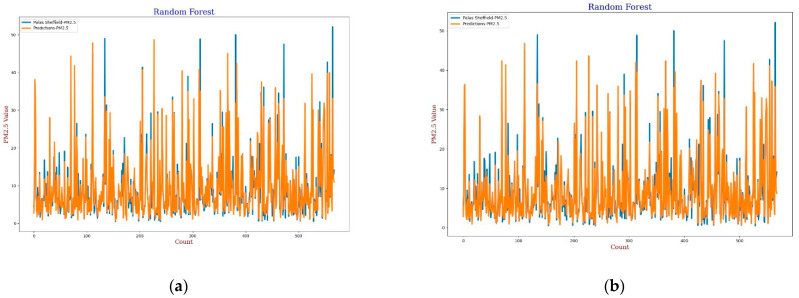
(**a**) RF calibration plot for *PM*_2.5_ using *AH*. Blue colour represents the reference data and orange colour represents the calibrated data; (**b**) RF calibration plot for *PM*_2.5_ using *RH*. Blue colour represents the reference data and orange colour represents the calibrated data.

**Figure 18:**
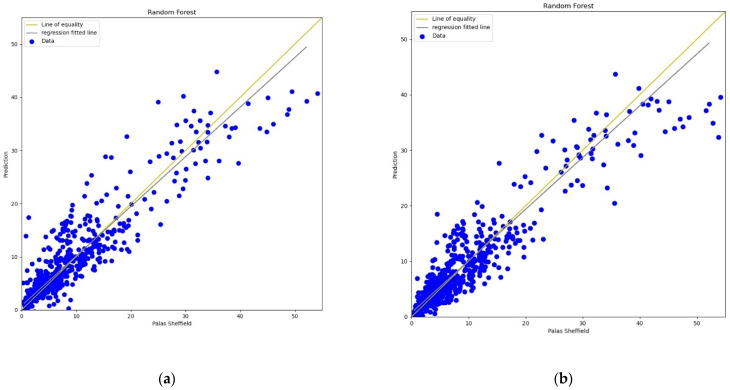
(**a**) RF calibration scatter plot for *PM*_2.5_ with the line of equality, regression fitted line, and the calibrated data using *AH*; (**b**) RF calibration scatter plot for *PM*_2.5_ with the line of equality, regression fitted line, and the calibrated data using *RH*.

**Figure 19:**
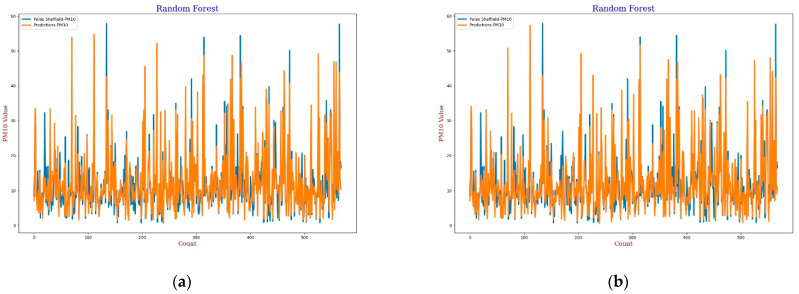
(**a**) RF calibration plot for *PM*_10_ using *AH*. Blue colour represents the reference data and orange colour represents the calibrated data; (**b**) RF calibration plot for *PM*_10_ using *RH*. Blue colour represents the reference data and orange colour represents the calibrated data.

**Figure 20:**
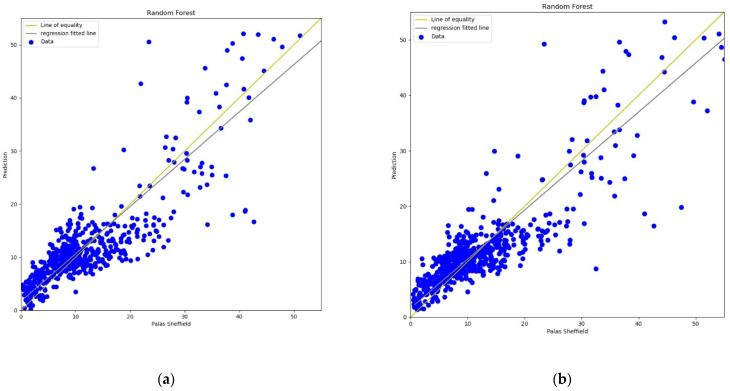
(**a**) RF calibration scatter plot for *PM*_10_ with the line of equality, regression fitted line, and the calibrated data using *AH*; (**b**) RF calibration scatter plot for *PM*_10_ with the line of equality, regression fitted line, and the calibrated data using *RH*.

The Random Forest (RF) model is a machine learning technique based on a combination of classification or regression trees which was first introduced by Breiman in 2001 [60]. In this experiment, 20 trees were used in the forest for calibration. The experimental results, lines, and scatter plots were analysed as completed for the previous three models. Figure 17, Figure 18, Figure 19 and Figure 20 show the lines and scatter plots obtained for *AH* and *RH* for both *PM*_2.5_ and *PM*_10_. The analysis of the plots shows similar results as the previous three models which supports the conclusion that *AH* gives better performance than *RH* for calibration.

## 4. Experimental Results, Analysis, and Discussion

Four calibration models were examined with the dataset of 1891 records for 1 month at the Sheffield site. Among the 1891 data, we divided it into a 70/30 ratio for training and testing data (number of training data = 1324 and number of testing data = 567) for all four models. For the comparative analysis, experimented results are summarised in Table 2 and Table 3 for *PM*_2.5_ and *PM*_10_, respectively. In both the tables, five fields are presented: *R*^2^ (Coefficient of Determination), *RMSE* (Root Mean Square Error), and *MAE* (Mean Absolute Error), Mean PMs’ reading from the reference station, and four calibration models. From Table 2, the comparative analysis for *PM*_2.5_, it can be observed that *R*^2^ values (Coefficient of Determination) for the four calibration models are nearly the same, ranging from 0.87 to 0.89 for the *AH* whereas there was more variance in *R*^2^ ranging from 0.84 to 0.88 when *RH* was used. Among four calibration models, the RF model has the highest *R*^2^ of both *AH* and *RH* cases. The next parameters that were compared are *RMSE* for both *AH* and *RH*. Analysing this, it was noted that the RF model has the lowest *RMSE* for both *AH* and *RH*, which tells us that it is able to fit the dataset the best out of the four calibration models. The next performance parameter that was compared is *MAE*. Comparing *MAE*, the RF model has less error than the other models. It has a nearly 47% improvement in errors in comparison with *MAE* for the MLR model.

The calibrated values from all four models were compared with the reference data. Mean values and standard deviations of reference data and calibrated models were compared. The comparative analysis showed that the mean values of MLR and CNN calibration models are closer than the other two models, MLP and RF, to the reference mean when *AH* was used. On the other hand, for the MLP and RF models mean values are closer than the MLR and CNN to the reference means when *RH* was used. Comparing the standard deviation, it was found that the RF model has the closest standard deviation values in both *AH* and *RH* cases to the reference standard deviation data. Similarly in Table 3, all four models are compared with each other for *PM*_10_. The comparative analysis reflected that there is a wider variance among the measured performance measures’ values for *AH* and *RH* for *PM*_10_ in comparison to *PM*_2.5_. From Table 3, it can be seen that the RF calibration model has fewer errors than the other three calibration models. It can also be observed that, for *MAE*, the RF model has a 25% improvement in *MAE* error measures than the other models. When comparing the mean readings, it is noted that the MLR model is closer to the reference mean and the MLP model is close to the reference standard deviation values for the Standard Deviation. From this comparative analysis of all these parameters, it was observed that the calibration models are performing better when *AH* was used as compared with *RH*. From the results, it was observed that the RF calibration model (*R*^2^ = 0.89, *RMSE* = 3.05, and *MAE* = 1.19) appeared as the best calibration output as compared with the other models for *PM*_2.5_. For the case of *PM*_10_, there was a variance in the performances of the different calibration models. The coefficient of determination of the RF (*R*^2^ = 0.83) model gives better results. However, it was also observed that the CNN model gives a better result (*R*^2^ = 0.81) with the use of *RH* for calibration, but *RMSE* and *MAE* are higher than RF as shown in Table 3 for *PM*_10_.

The experimental setup in our use case involving LCS-based AQ monitoring, and methodology covering the sensor selection and calibration, are transferable to similar applications across different domains. This methodology has the potential to be considered with its key success factors to make any LCS-based application kit design an innovative solution. This proposed methodology opens the door for efficient and effective practices for LCS-based applications.

## 5. Conclusions and Future Work

LCSs give an alternative solution against the high-cost sensors used for various measurement and monitoring purposes as they are compact in size and low-cost. However, it has been also observed that the use of LCS-based applications is challenging due to inconsistency in standard and different alternatives when measuring the same components. Additionally, the LCS has appeared as a multipurpose tool and the system is easy to configure, however, it is difficult to select the right LCS for a specific task due to the diversity of LCSs available in the market with similar configurations. Even the sensor selection process has been carefully prepared but it needs to anticipate the possible obstacles such as delay in supply and different measurement units for the same purpose of measurement. Furthermore, some of the LCSs’ parameters can directly impact sensor performance and data reliability, for example, meteorological parameters make the sensor selection and calibration process even harder. By examining the experimental outcomes from different sensors, we found that there have been different environmental responses of individual sensors. We also observed that there have been consistency issues among the sensors from the same manufacturer which appear as a challenging factor in deciding on a sensor during the sensor selection stage. From this study, we also found that the consistency and sensitivity of individual LCSs to environmental factors including temperature and humidity need to be analysed before applying the calibration.

The confidence in data from LCSs is lower as it required calibration. Therefore, building a device using LCSs is challenging and required some methodology on sensor selection and data processing. Our data-driven approaches provide a methodology that can help to build LCS-based devices from the sensor selection process and their calibration. To validate this methodology, experimental analysis was performed with different candidate sensors along with data collection. The data-driven approach provides a methodology to enhance data quality. Four widely used calibration models were applied for the LCS-based AQ device to analyse, and hence comparison was performed among the calibration models against the high-cost monitoring station data. Calibration parameters were established at pre-defined locations with a high-cost reference station. This calibration process can also be transferable to other reference stations and sensors depending on the sensor types and their application.

The comparison among four commonly used calibration methods was presented to determine the best-suited calibration model in our use case study. In addition, from the analysis, it was also observed that *AH* has better performance than *RH* in the sensor calibration. Among the four models, the RF model appeared as the best model for the calibration of LCSs. To bring more confidence to this work, the calibrated LCS devices will be deployed across the different regions in Bradford, UK, as a use case study for 3–6 months in the near future. The data coming from calibrated LCS devices will be analysed against the reference values over a longer duration to analyse the drift in LCS performance. Additionally, techniques and methodologies for re-calibration will be further explored to enhance the data quality of LCS-based monitoring systems.

The presented methodology did not include any uncertainty analysis as the observation was applied for only a short duration for data analysis for sensor selection and calibration. However, for any LCS-based applications, an acceptable uncertainty needs to be defined during the measurements. Additionally, in this study, the PM sensors were only analysed and calibrated. This appears as a limitation of this study as there are gas sensors that have also been used to monitor gas as a pollutant in AQ. As future work, a longer period of observation and data analysis needs to be applied to add additional confidence and to reduce the uncertainty in monitored data from LCSs. Acceptable uncertainty is required to ensure the measurement uncertainties are lower and are sufficient to make the calibration results valid. Acceptable uncertainty also ensures the uncertainty in data does not affect the LCS-based monitoring objectives. In addition, the same principles can be applied to LCS-based gas sensors.

## Figures and Tables

**Table 1:** List of candidate sensors and their details.

Candidate Sensor Name	Description	Sensor Specification	Image
BME680	This sensor can measure temperature, humidity, barometric pressure, and VOC gas.	Temp in Celsius (°C); Humidity—%; barometric pressure—hPa	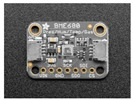
CJMCU-811	This sensor can be used for detecting eCO_2_, VOC gases. It is a digital gas sensor integrated CCS801 sensor and 8-bit analogue-to-digital converter (ADC).	eCO_2_ in ppm, VOC gases in ppb.	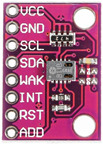
SGP-30	This gas sensor is mainly used to monitor eCO_2_ and TVOC.	eCO_2_ in ppm, VOC gases in ppb.	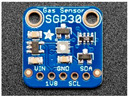
Envio+	This pHAT is a collection of multiple sensors such as BME280 which can measure temperature, humidity, and pressure, MICS6814 analogue gas sensor is responsible for measuring CO, NO_2_, and ammonia (NH_3_) and LTR-559 is light and proximity sensor. Additionally, it has a built-in ADS1015 analogue-to-digital convertor and 0.96 “colour LCD for display”.	BME280: temperature (°C); pressure (hPa), humidity (%) LTR-559 light and proximity sensor MICS6814 analogue gas sensor (CO, NO_2_, NH_3_)	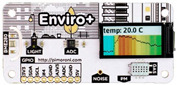
SDS011	This sensor is used to measure *PM*_2.5_ and *PM*_10_ air pollutants. This sensor is an infrared-based laser sensor and has a fan to provide self-airflow.	*PM*_2.5_: ug/m^3^ *PM*_10_: ug/m^3^	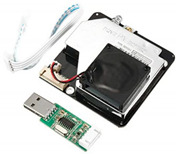
PMS5003	It is used to measure *PM*_1_, *PM*_2.5_, and *PM*_10_.	*PM*_2.5_: ug/m^3^ *PM*_10_: ug/m^3^	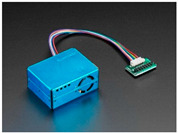
OPC-R1	This sensor is used to detect *PM*_1_, *PM*_2.5_, and *PM*_10_ with the help of laser scattering technology.	*PM*_2.5_: ug/m^3^ *PM*_10_: ug/m^3^	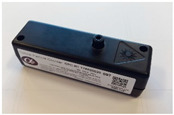
MQ-2	This gas sensor is mainly used to detect CO, methane, butane, LPG, smoke.	LPG and propane—200–5000 ppm Butane—300–5000 ppm Methane—5000–20,000 ppm Hydrogen—300–5000 ppm Alcohol—100–2000 ppm	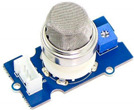

**Table 2:** Statistical performance measures analysis for *PM*_2.5_.

Model	*R* ^2^		*RMSE*		*MAE*		Mean Reading (After Calibration) Reference Mean = 9.32 μg/m^3^		Standard Deviation (After Calibration) Reference Standard = 9.26 μg/m^3^	
	*AH*	*RH*	*AH*	*RH*	*AH*	*RH*	*AH*	*RH*	*AH*	*RH*
MLR	0.87	0.84	3.32	3.65	2.19	2.58	9.36	9.86	8.72	9.13
MLP	0.88	0.85	3.20	3.48	2.13	2.18	9.60	8.10	9.08	7.94
CNN	0.89	0.88	3.07	3.65	2.01	2.30	9.26	10.29	8.32	9.50
RF	0.89	0.88	3.05	3.07	1.19	1.86	9.75	9.67	9.05	9.02

**Table 3:** Statistical performance measures analysis for *PM*_10_.

Model	*R* ^2^		*RMSE*		*MAE*		Mean Reading (After Calibration) Reference Mean = 12.24 μg/m^3^		Standard Deviation (After Calibration) Reference Standard = 9.75 μg/m^3^	
	*AH*	*RH*	*AH*	*RH*	*AH*	*RH*	*AH*	*RH*	*AH*	*RH*
MLR	0.79	0.75	5.28	4.95	3.69	3.53	12.39	12.52	9.10	8.81
MLP	0.81	0.78	4.43	4.68	3.13	3.26	12.64	12.35	9.55	9.01
CNN	0.80	0.81	4.42	4.71	3.04	3.19	12.45	12.10	9.15	9.09
RF	0.83	0.83	4.03	4.05	2.78	2.77	12.64	12.45	9.43	9.38

## Data Availability

Not Applicable.
